# PP12 Projecting The Potential Impact Of Disease-Modifying Therapies For Alzheimer’s Disease On The Carbon Emissions From Hospital Bed-Days in the UK

**DOI:** 10.1017/S0266462324001855

**Published:** 2025-01-07

**Authors:** Dominic Trepel, Sophie Edwards, Craig Ritchie, Julie Hviid Hahn-Pedersen, Jamie Kettle, Mei Sum Chan, Benjamin D Bray, Alice Clark, Milana Ivkovic, Christian Ahmad Wichmann, Marc Evans

## Abstract

**Introduction:**

Disease-modifying therapies (DMTs) for Alzheimer’s disease (AD) are emerging treatment options. This study aimed to estimate the potential health system and associated environmental impacts of DMTs by modeling future bed-days and carbon dioxide equivalent (CO_2_e) emissions for the UK population under various scenarios for access to and efficacy of DMTs.

**Methods:**

A cohort Markov model was developed to predict the UK population distribution from 2020 to 2040 across five health states—cognitively unimpaired and four stages of AD (mild cognitive impairment, and mild, moderate, severe dementia). These distributions were estimated using national population projections, AD prevalence data, and stage-specific transition rates. Annual bed-days per person for each state and associated CO_2_e emissions from published literature were applied to estimate total bed-days and emissions. Modeled scenarios combined ranges of DMT efficacy estimates (20 to 30%) and access levels (25 to 58% eligible patients receiving treatment) elicited from expert opinion to explore the extent of potential DMT impacts.

**Results:**

Without DMT access, annual bed-days across the four AD stages were projected to increase from 5.5 million to 8.6 million from 2020 to 2040, with cumulative bed-days totaling 140 million. Associated annual emissions increased from 0.7 Mt to 1.1 Mt CO_2_e, reaching 17 Mt CO_2_e cumulatively from 2020 to 2040. Under the various high-access (58% eligible patients treated) DMT efficacy scenarios, relative to no DMT access, annual reductions of 430 thousand to 650 thousand bed-days and 54 kt to 81 kt CO_2_e were estimated by 2040, and cumulative emissions decreased by 419 kt to 633 kt CO_2_e. Decreasing DMT access to 25 percent, assuming 25 percent DMT efficacy, reduced annual bed-days by 230 thousand by 2040, and annual emission savings decreased to 29 kt CO_2_e.

**Conclusions:**

DMTs for AD may contribute to efforts by healthcare systems to reduce the carbon emissions from hospital inpatient care. Environmental sustainability should be considered as part of a holistic value proposition when assessing the benefits of new medicines.

